# Loading calcium fluorescent probes into protoplasts to detect calcium in the flesh tissue cells of *Malus domestica*

**DOI:** 10.1038/s41438-020-0315-3

**Published:** 2020-06-01

**Authors:** Lina Qiu, Yongzhang Wang, Haiyong Qu

**Affiliations:** College of Horticulture, Qingdao Agricultural University, Qingdao City, China

**Keywords:** Fluorescence spectroscopy, Single-cell imaging, Plant physiology

## Abstract

Cytosolic Ca^2+^ plays a key role in signal transduction in plants. Calcium imaging is the most common approach to studying dynamic changes in the cytoplasmic Ca^2+^ content. Here, we used mature ‘Fuji’ apples (*Malus pumila Mill*.) to obtain viable protoplasts from flesh tissue cells by enzymatic hydrolysis; then, three small-molecule fluorescent probes (fluo-8/AM, fluo-4/AM, and rhod-2/AM) were loaded into the protoplasts. All three Ca^2+^ fluorescent probes successfully entered the cytoplasm but did not enter the vacuole. Both the Ca^2+^ chelator (EGTA) and Ca^2+^ channel blocker (La^3+^) reduced the fluorescence intensity in the cytoplasm. The calcium ionophore A23187 increased the fluorescence intensity in the cytoplasm at 5 µmol/L but decreased it at 50 µmol/L. Additionally, A23187 reversed the fluorescence intensity in the cytoplasm, which was decreased by La^3+^. Ionomycin is also a calcium ionophore that can increase the fluorescence intensity of calcium in the cytoplasm. These results suggest that small-molecule Ca^2+^ fluorescent probes can be used to detect changes in cytosolic calcium levels in the cells of fruit flesh tissue. In addition, the optimum concentration of fluo-8/AM was determined to be 5 µmol/L. This was the first time that protoplasts have been isolated from apple flesh tissue cells and small-molecule fluorescent probes have been used to detect calcium in the cytoplasm of flesh tissue cells. This study provides a new method to study calcium signal transduction in fruit flesh tissue.

## Introduction

Calcium influences many fruit qualities^1,2^; it affects fruit firmness, sugar content, storage period, and physiological disorders during storage^3,4^. Thus, a low calcium content reduces fruit firmness and shortens the length of the storage period. A disorder in cell calcium metabolism can cause apple bitter pit^5^, brown spot disease^6^, and cock spot in *Pyrus serotina*^7^. In vegetable crops, tomato umbilical rot is also caused by a calcium metabolism disorder^8^. These physiological disorders severely degrade fruit quality and cause serious economic losses to growers^2,9^. Therefore, growers often supplement fruit with calcium. Spraying calcium early during fruit growth and soaking in calcium at postharvest can improve fruit firmness^10^, reduce the incidence of bitter pit^11^ and cock spot^7^, delay fruit senescence and softening, and extend the storage period^2^.

However, calcium supplementation is not always effective. Studies have shown that calcium spraying or soaking does not always increase fruit firmness^3^. Spraying calcium on the fruit skin surface is not an effective way to prevent litchi fruit cracking^12^, nor can it reduce the incidence of apple bitter pit, as was shown in a study in which calcium spraying was repeated five times over the entire apple fruit growth period^13^. Moreover, other studies have shown that the calcium content of bitter-pit fruit is not lower than that of healthy fruit^3,14^. The main reason for these conflicting results is that the role of calcium in flesh tissue cells is not clear^15^. In the cytoplasm, Ca^2+^ acts as a signaling ion that mediates a variety of cell growth and development processes^16^.

Calcium imaging has been demonstrated to be a powerful method for observing the dynamic changes in intracellular Ca^2+^ in living cells with good spatial and temporal resolution^17,18^. At present, there are two methods for intracellular calcium imaging in living cells: one uses small-molecule fluorescent probes, and the other uses FRET (Förster Resonance Energy Transfer)—based genetically encoded sensors (GECIs)^19^. Although GECIs have many advantages, a stable transgenic system for fruit trees is difficult to establish, and the growth period of fruit trees is inconveniently long;^20^ furthermore, the weak fluorescence of single cells is not conducive to overcoming background noise, and the method is susceptible to interference from endogenous calmodulin and other shortcomings^21^.

Small-molecule fluorescent indicators, such as fluo-4/AM and fluo-8/AM, show Ca^2+^-specific selectivity and are noninvasively loaded by esterification incubation, which is flexible, rapid, and not cytotoxic^22^. Fluo-4/AM was successfully loaded into the pollen tube of *Pyrus pyrifolia*^20^ and petunia^23^ as well as into the guard cells^24^ and root hairs^25,26^ of Arabidopsis. Fluo-8/AM can also be used to detect dynamic calcium in guard cells^27^.

At present, the study of plant calcium imaging mainly focuses on pollen tubes, root hairs, and guard cells. There are few reports on calcium fluorescence staining of flesh cells. Calcium may be the most important mineral determining the quality of fruits, especially apples and pears, because they are commonly stored for long periods of time^28^. Moreover, apples are economically important worldwide and a healthy food^29^. Today, ‘Fuji’ apples are one of the most popular sweet apple varieties in the world and are commercially grown in Japan, China, the United States, and Australia. Especially in China, ‘Fuji’ is the main planting variety, and its yield and cultivated area account for more than 60% of the total apple production and total cultivated area^30^. Here, we first obtained viable protoplasts from ‘Fuji’ apple flesh tissue cells and then detected Ca^2+^ in the cytoplasm with a small-molecule calcium fluorescent reagent.

## Results

### Flesh tissue staining with Ca^2+^ fluorescence

**Fig. 1:**
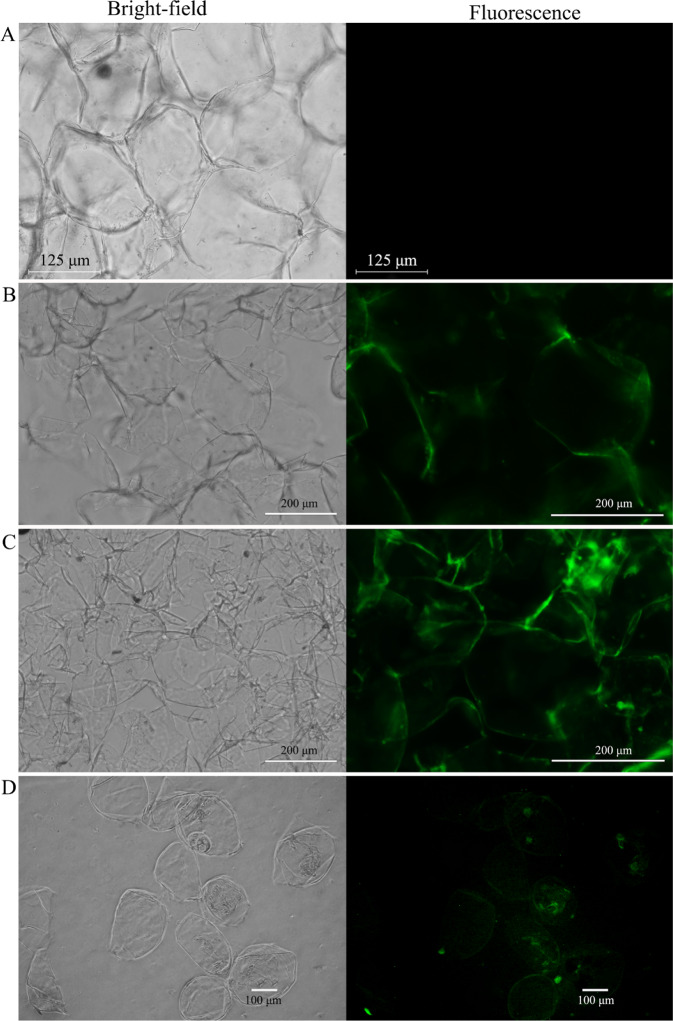
Loading of fluo-8/AM into flesh tissue cells. **a** Slice of flesh tissue that was not loaded with fluo-8/AM. **b** Flesh tissue was cut by a cryostat and then fluorescently stained with fluo-8/AM. **c** Flesh tissue was cut by hand and then fluorescently stained with fluo-8/AM. **d** Fluo-8/AM loaded into single flesh cells

Apple fruit flesh was manually cut as thin as possible with a surgical knife. The slices did not display fluorescence prior to loading with fluo-8/AM (Fig. 1a), which indicated that the flesh tissue cells were not self-fluorescing. A cryostat was then used to cut the flesh tissue to a thickness of 80 µm (i.e., a single cell layer), and then the tissue was stained with fluo-8/AM at 37 °C for 30 min. In this case, fluorescence was observed only around the cell, i.e., against the cell wall (Fig. 1b). Although the manually cut flesh tissue was sliced as thin as possible, it was difficult to ensure that the slices were single-cell layers. Nonetheless, after loading fluo-8/AM into the slices, the staining results from the hand-sliced tissue were consistent with those from the slices cut with the cryostat and only showed fluorescence at the cell edges (Fig. 1c). Then, single flesh cells were obtained by enzymatic hydrolysis. Despite some fluorescence at the edge of the cells after fluo-8/AM loading, the fluorescence intensity was very low (Fig. 1d). As there is a large vacuole in the center of the flesh cell with the cytoplasm squeezed around the cell (Supplementary Fig. S1A,B), fluorescence appeared around the cell. The cell wall is a pool of Ca^2+^ that can also be combined with a Ca^2+^ fluorescence indicator;^31^ thus, it was difficult to determine whether fluorescence was from the cell wall or from the cytoplasm.

### Protoplast viability assay

**Fig. 2:**
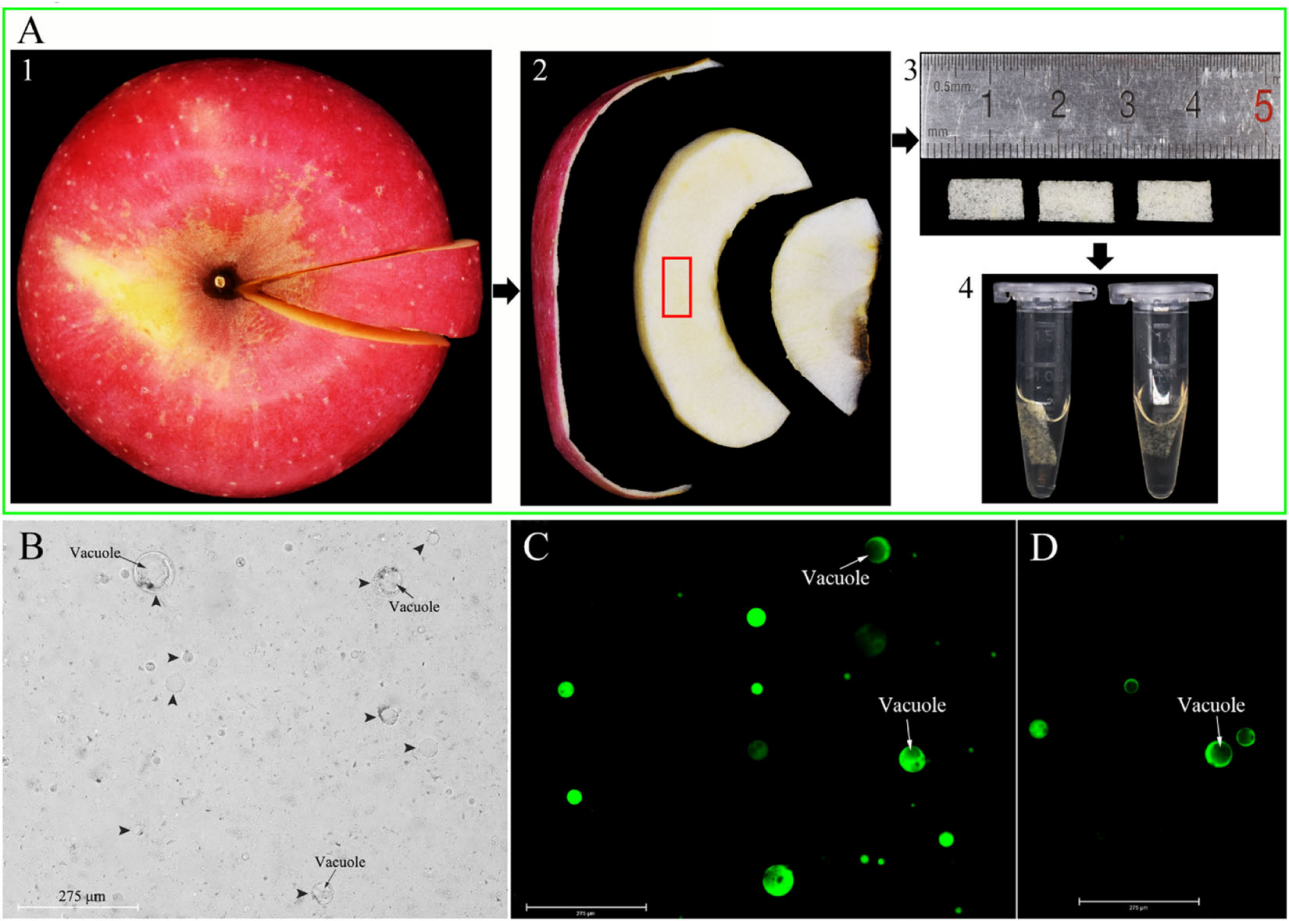
Protoplasts were obtained by enzymatic hydrolysis. **a** (1) A healthy and mature apple was selected from which a piece was cut; (2) a sample of the mesocarp was cut; (3) the mesocarp cube was cut to 10 × 5 × 1 mm^3^ (4) mesocarp cubes were placed in a centrifuge tube containing the enzyme solution. **b** Protoplasts of different diameters. Arrowheads point to the protoplast, and arrows point to the vacuole in the protoplast. **c** Protoplasts were stained with FDA. **d** Protoplasts were incubated at 37 °C for 30 min and then stained with FDA

We used an enzymatic method to obtain protoplasts from individual apple flesh cells (Fig. 2a and Methods). Some protoplasts had vacuoles, while others did not (Fig. 2b). We measured the diameter of 50 protoplasts and found an average diameter of 48 µm (Supplementary Fig. S2). Protoplasts were stained with FDA for 5 min and showed fluorescence in the cytoplasm, indicating that the isolated protoplasts were viable (Fig. 2c). Protoplasts were stored at 37 °C for 30 min and then stained with FDA; they were still fluorescent (Fig. 2d), indicating that the high temperature (37 °C) did not affect their viability.

### Protoplast calcium ion fluorescence staining

**Fig. 3:**
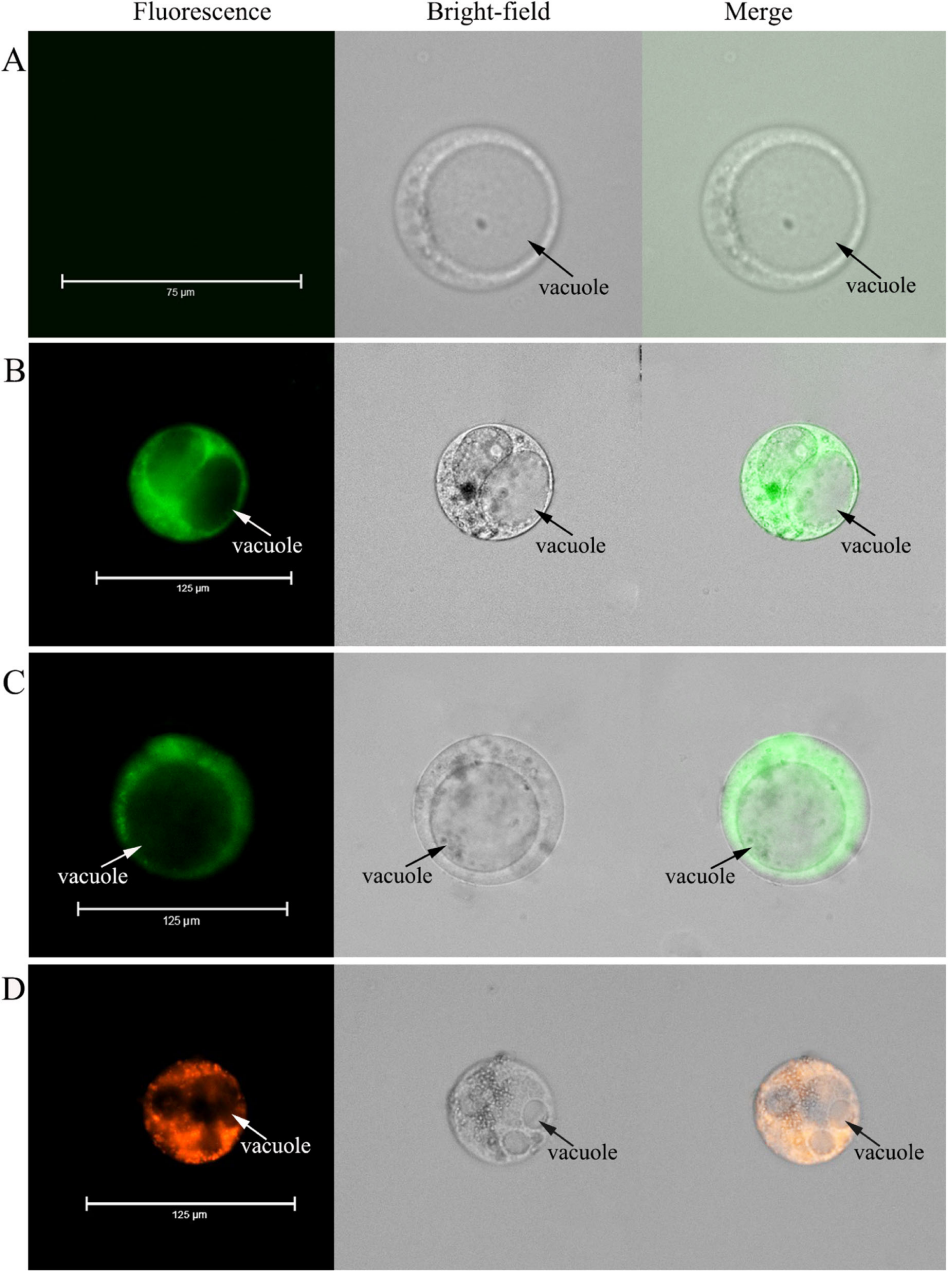
Loading of three kinds of Ca^2+^ fluorescent dyes into the protoplasts from flesh cells. **a** Intact protoplast without any loaded fluorescent probes. **b** Protoplast loaded with fluo-8/AM. **c** Protoplast loaded with fluo-4/AM. **d** Protoplast loaded with rhod-2/AM

We loaded different small fluorescent indicators into the protoplasts (Methods) to measure cytoplasmic Ca^2+^. Protoplasts showed no fluorescence when no Ca^2+^ fluorescent indicator was loaded into them (Fig. 3a). On the other hand, when either fluo-8/AM or fluo-4/AM were loaded into the protoplasts, the cytoplasm was fluorescent, but the vacuole was not (Fig. 3b, c). Furthermore, when rhod-2/AM was loaded into the protoplast, there was still fluorescence in the cytoplasm and none in the vacuoles (Fig. 3d). However, the results of rhod-2 staining were different from those of fluo-4/AM or fluo-8/AM staining. The fluorescence in the protoplasts was granular (Fig. 3d), as rhod-2/AM entered the mitochondria and stained Ca^2+^ within the mitochondria^32^. These results suggested that fluo-4/AM and fluo-8/AM successfully stained Ca^2+^ in the cytoplasm and that there was no compartmentalization in either case^33^.

### Effects of different concentrations of fluo-8/AM on the fluorescence intensity of calcium in protoplasts

**Fig. 4:**
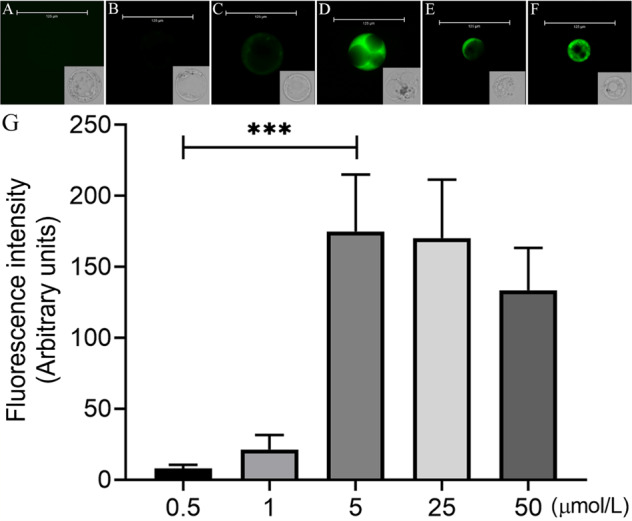
Loading of different concentrations of fluo-8/AM into flesh cell protoplasts. **a** Control protoplast not loaded with fluo-8/AM. **b** 0.5 µmol/L. **c** 1 µmol/L. **d** 5 µmol/L. **e** 25 µmol/L. **f** 50 µmol/L. **g** Statistical analysis of fluorescence intensity in the protoplasts. As there was no self-fluorescence phenomenon in the control, there was no statistical control of fluorescence density. *** Indicates a significant difference (*P* < 0.001, Student’s *t*-test). Ver*t*ical bars indicate ± SE. Each data point represents the mean of 10 protoplasts. **a**–**f** Representative fluorescence images of protoplasts

We used fluo-8/AM as a reference reagent to detect the effect of different concentrations of a fluorescent reagent on the fluorescence intensity of calcium in the cytoplasm. When the concentration of fluo-8/AM increased from 0.1 to 5 μmol/L, the fluorescence intensity of calcium ions gradually increased (Fig. 4a–d). Particularly from 1 to 5 μmol/L, the fluorescence intensity increased significantly (*P* < 0.001) (Fig. 4g). However, when fluo-8/AM exceeded 5 μmol/L, the fluorescence intensity of calcium decreased (Fig. 4e, f), but the difference was not significant *(p* > 0.05) (Fig. 4g). We calculated the diameters of protoplasts after different concentrations of fluo-8/AM staining. When the concentration of fluo-8/AM exceeded 5 μmol/L, the diameter of the protoplasts decreased (Supplementary Fig. S3), mainly due to the increase in the ratio of protoplast shrinkage or breakage (Supplementary Fig. S4). Although there are no specific reports on the effect of fluo-8/AM on protoplast membranes, we believe that the high concentration of fluo-8/AM had a destructive effect on the protoplast membrane. Therefore, we suggest that the optimal concentration of fluo-8/AM for calcium fluorescence staining in protoplasts of flesh cells is 5 μmol/L.

### Reagents that affect the response of Ca^2+^ fluorescence in protoplasts

**Fig. 5:**
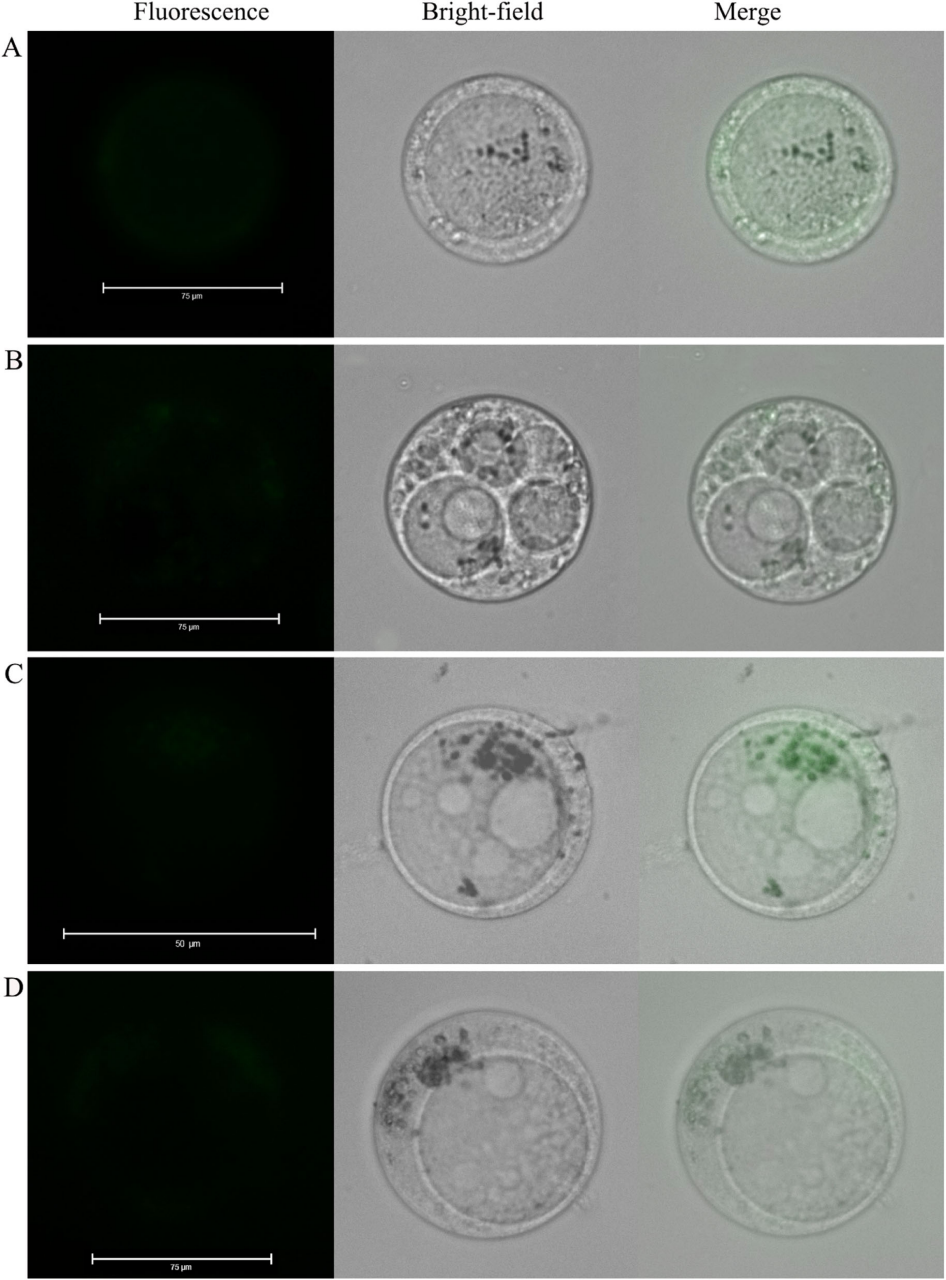
Effect of different concentrations of EGTA or La^3+^ on Ca^2+^ concentration in protoplasts. **a** Protoplast after loading with fluo-8/AM, followed by the addition of 1 mmol/L EGTA, **b** 10 mmol/L EGTA, **c** 10 µmol/L La^3+^, or **d** 100 µmol/L La^3+^

**Fig. 6:**
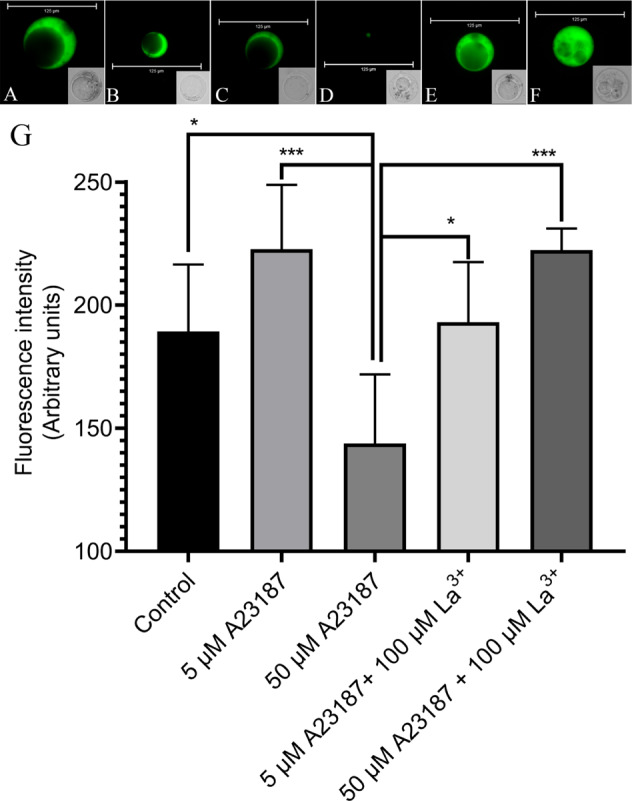
Effect of A23187 on Ca^2+^ concentration in protoplasts. **a** Control protoplast loaded with fluo-8/AM. **b** Protoplast after loading with fluo-8/AM, followed by the addition of 5 µmol/L A23187 or **c** 50 µmol/L A23187; **d** protoplast after loading with fluo-8/AM, followed by the addition of 100 µmol/L La^3+^. **e** Protoplast after loading with fluo-8/AM, followed by the addition of 5 µmol/L A23187 and 100 µmol/L La^3+^. **f** 50 µmol/L A23187 and 100 µmol/L La^3+^. **g** Statistical analysis of fluorescence intensity in the protoplasts. *** Indicates a significant difference (*P* < 0.001, Student’s *t*-test). * Indicates a significant difference (*P* < 0.05, Student’s *t*-test). Vertical bars indicate ± SE. Each data point represents the mean of 10 protoplasts. **a**–**f** Representative fluorescence images of protoplasts

**Fig. 7:**
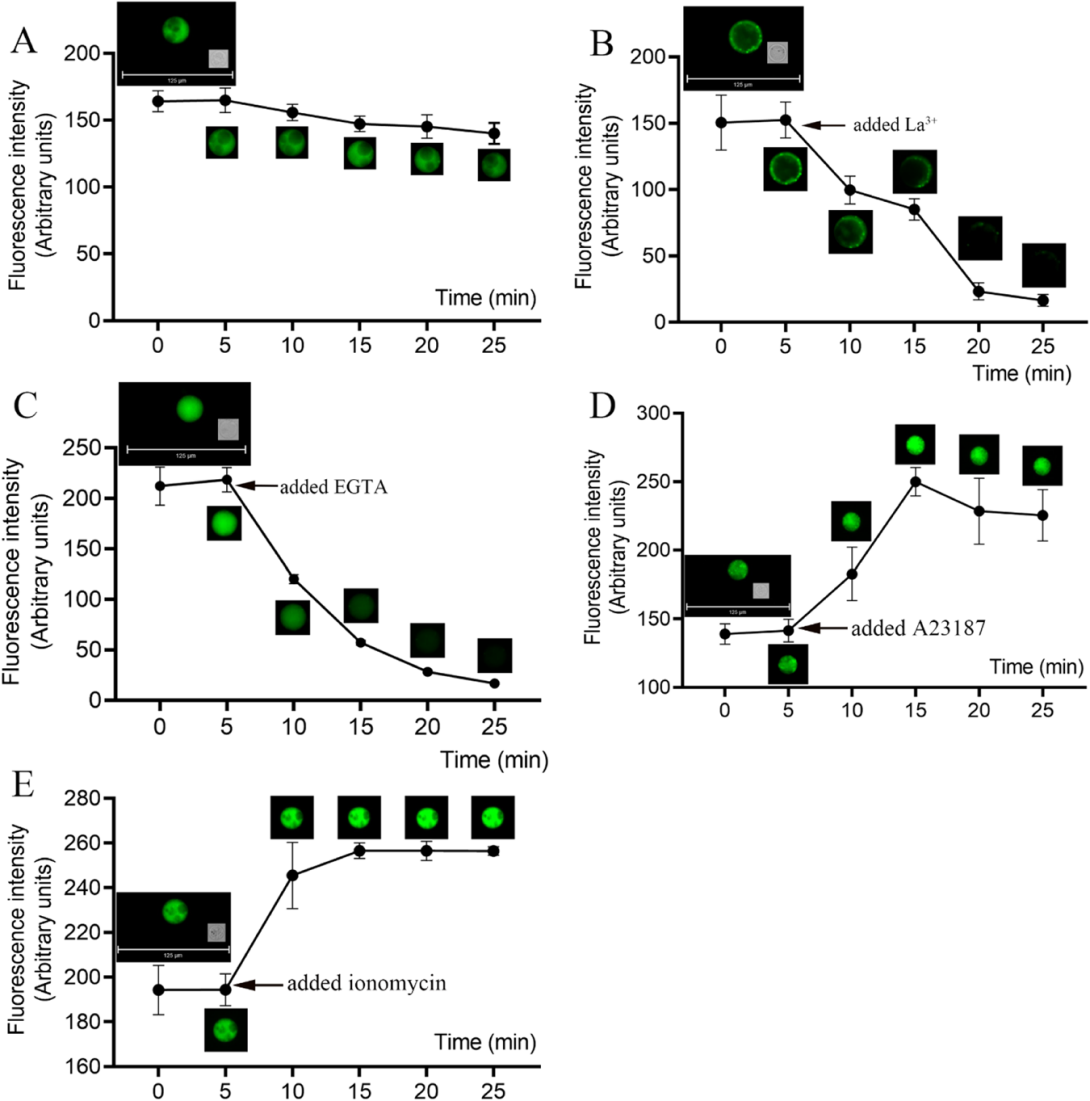
Fluorescence changes in Ca^2+^ in the same protoplast within 25 min. Pictures were taken every 5 min. **a** Protoplast loaded with fluo-8/AM (Control). **b** The calcium ion fluorescence was observed for 5 min, and then 100 µmol/L La^3+^ was added. **c** The calcium ion fluorescence was observed for 5 min, and then 10 mmol/L EGTA was added. **d** Calcium ion fluorescence was observed for 5 min, and then 5 µmol/L A23187 was added. **e** The calcium ion fluorescence was observed for 5 min, and then 1 µmol/L ionomycin was added. Vertical bars indicate ± SE. Each data point represents the mean of three protoplasts. **a**–**e** Representative fluorescence images of protoplasts

A Ca^2+^ chelator (EGTA)^34^ was added to the protoplasts with fluo-8/AM at final concentrations of 1 mmol/L or 10 mmol/L. EGTA is a chelating agent for Ca^2+^ that can significantly reduce fluorescence intensity (Fig. 5a, b). Additionally, La^3+^, which is a calcium-ion channel blocker on the cell membrane^35^, was added to protoplasts during fluo-8/AM loading; La^3+^ could also significantly decrease fluorescence intensity, regardless of whether the final concentration was 10 or 100 µmol/L (Fig. 5c, d). Protoplasts were treated with calcium ionophore A23187^36,37^ to a final concentration of 5 µmol/L when fluo-8/AM was loaded into the protoplasts. This treatment might have increased the fluorescence intensity in the cytoplasm relative to that of the controls (Fig. 6a, b, g); however, contrary to expectation, A23187 significantly decreased fluorescence intensity when it reached a final concentration of 50 µmol/L (Fig. 6a, c, g), likely owing to La^3+^-mediated reduction of fluorescence intensity (Fig. 6d–g). We observed changes in Ca^2+^ fluorescence in the same protoplast. The protoplasts without any treatment (control) showed a slight decrease in fluorescence intensity within 25 min (Fig. 7a), and the change was not significant. La^3+^ and EGTA reduced the Ca^2+^ fluorescence intensity within 5 min and completely quenched the fluorescence within 25 min (Fig. 7b, c). A23187 increased the Ca^2+^ fluorescence intensity in protoplasts within 5 min, but the fluorescence intensity reached a peak at 10 min and then decreased slightly (Fig. 7d). We also used another calcium ionophore (ionomycin) to increase the fluorescence intensity of the protoplasts, and the fluorescence intensity did not decay within 25 min (Fig. 7e). In addition, after La^3+^ reduced the fluorescence of the same protoplast, supplementation with A23187 increased the calcium fluorescence intensity (Supplementary Fig. S5). This result further demonstrated that fluo-8/AM can stain Ca^2+^ in the cytoplasm and show dynamic changes in the Ca^2+^ content. Calcium in the cytoplasm is maintained by an influx of extracellular calcium.

## Discussion

Calcium imaging is a useful technique for studying the roles of Ca^2+^ in living cells^18^. In plants for which stable transgenic systems are difficult to establish, small-molecule calcium fluorescent indicators are commonly used for cellular calcium imaging^20^. We used enzymatic hydrolysis to obtain viable apple flesh protoplasts and then loaded Ca^2+^ fluorescent probes into the protoplasts for cytoplasmic calcium imaging. Ca^2+^ in the cytoplasm plays an important role in signaling^38^. Under CO_2_ and high pressure, strawberry firmness was shown to increase, but it was suggested that such an increase would be delayed if intracellular Ca^2+^ efflux was inhibited^39^. Therefore, calcium imaging in the cytoplasm of flesh cells provides a powerful tool for studying the physiological role of calcium in fruit growth and development^40^.

Small-molecule calcium fluorescent probes cannot cross the membrane into the cell. Therefore, the indicator should be added with an acetoxymethyl (AM) ester, and the dye should be made neutral so that it can cross the cell membrane^41^. However, esterases on the cell membrane can cleave AM groups and prevent the indicator from entering the cell^20^. There are a variety of methods for loading fluorescent probes into plant cells. Zhang et al. (1988)^42^ loaded fluo-3/AM at a low temperature (4 °C) so that it would enter the root tip cells. Similarly, Qu et al. (2012)^43^ used fluo-3/AM, but at a high temperature (37 °C), to image the inside of pollen tubes. These methods aimed to reduce the activity of esterases on the cell membrane. To overcome the barrier of the cell membrane, the microinjection method has been used to directly inject the fluorescent probes into the cell. The cell wall is a pool of Ca^2+^ that interferes with the fluorescent intensity of Ca^2+^ in the cytoplasm when loaded with a fluorescent probe^31^. To avoid this interference, the microinjection method is also used to avoid the cell wall^44^. However, this method is very complicated and slow^31,33^. In a previous study, we used cell lysates to slightly degrade the cell membrane without reducing cell viability and allowed the calcium fluorescent probe to enter the pollen tube^20^. In the present study, we successfully removed the cell wall of flesh cells by enzymatic hydrolysis and loaded fluorescent probes into protoplasts at a high temperature (37 °C). This method did not affect the viability of the protoplasts. Additionally, we loaded fluo-8/AM into the protoplasts at low temperature (4 °C) but failed to stain cytoplasmic Ca^2+^ (Supplementary Fig. S5).

Using small-molecule calcium fluorescent probes to stain intracellular calcium is not as advantageous as using GECIs. Indeed, a major drawback of the former method is that once the probe enters the cell, it is subject to compartmentalization^33^. In other words, it is easy for the probe to accumulate in the vacuole. In the cytoplasm, calcium concentrations are only 100–200 nmol/L, which is much lower than the concentration in the vacuole^45^, where it ranges from 0.1 to 10 mmol/L^45^. Nonetheless, the vacuole showed almost no fluorescence in this study, a finding that suggests that none of the three calcium fluorescent probes used were compartmentalized in the protoplasts. In addition, calcium fluorescence was uniformly distributed in protoplasts without vacuoles (Supplementary Fig. S6).

Intracellular Ca^2+^ fluorescence intensity can be reduced by lowering the Ca^2+^ concentration^46^. In the present study, the Ca^2+^ chelator EGTA seriously decreased the fluorescence intensity of calcium. In turn, La^3+^ blocks calcium channels on the cell membrane; however, the results of La^3+^ treatment in this study regarding the effects of cytoplasmic calcium were inconsistent. It has been suggested that a significant reduction in cytoplasmic Ca^2+^ concentration might be due to the inhibition of extracellular calcium influx^47^. However, some studies suggest that although La^3+^ inhibits extracellular calcium influx, it can also cause stored intracellular calcium to be released, thereby increasing Ca^2+^ cytoplasmic concentration^48^. In the experiments reported herein, La^3+^ significantly reduced Ca^2+^ concentration in the cytoplasm of the flesh cells, and calcium ionophore A23187 reversed the inhibitory effect of La^3+^ in vitro.

Babcock et al. (1976) studied the effects of A23187 on Ca^2+^ in bovine epididymal spermatozoa. They suggested that the effects of A23187 on the intracellular Ca^2+^ concentration were highly complex^36^. A23187 promotes Ca^2+^ efflux at low concentrations, while it promotes intracellular accumulation of Ca^2+^ at high concentrations^36^. However, 0.1 µmol/L A23187 inhibited the absorption of Ca^2+^ in the cardiac sarcoplasmic reticulum and only promoted it at 1 nmol/L^49^. Other studies have suggested that the effects of A23187 on Ca^2+^ flux depend on extracellular Ca^2+^ concentration^50^. Thus, for example, A23187 increased the Ca^2+^ concentration in hepatic stellate cells at 30 µmol/L but caused cell apoptosis^51^. Consistently, in the present study, A23187 increased the Ca^2+^ concentration in the cytoplasm at low concentrations (5 µmol/L) but decreased it at high concentrations (50 µmol/L). As a calcium carrier^52^, it has been reported that ionomycin can increase the concentration of calcium in the cytoplasm^53^, and in this study, ionomycin increased the fluorescence density of calcium in the cytoplasm of apple pulp cells.

Postharvest softening of apples is a very serious problem for apple growers in many countries^4^. Softening of apple fruits is generally considered an undesirable ripening process because firmer apples tend to have more juice and are crisper crunchier and less mealy than softer apples^54^. Ca^2+^ plays a key role in fruit firmness^14^. Therefore, calcium is added to the fruit before or after harvesting to increase the firmness of the fruit or reduce the speed of fruit softening^55^. We supplemented Ca^2+^ and EGTA through the fruit stalk 15 days before the harvest of ‘Golden Del. Reinders’ apple. Exogenous Ca^2+^ could increase the firmness of the fruit, while EGTA reduced the firmness of the fruit (Supplementary Fig. S8A). Exogenous Ca^2+^ also increased the cytosolic calcium concentration of fruit cells, while EGTA decreased the cytosolic concentration (Supplementary Fig. S8B,C). The results suggested that there was a positive correlation between Ca^2+^ concentration in the cytoplasm and fruit firmness. The rapid physiological degradation after harvest greatly reduced the quality and marketability of cassava (*Manihot esculenta Crantz*). Exogenous Ca^2+^ reduces postharvest physiological deterioration by increasing endogenous levels of Ca^2+^ and inducing the expression of genes related to melatonin biosynthesis after harvest. These effects are reversed by the exogenous application of EGTA^56^. Our findings support this result. However, Deell et al. (2001) suggested that the application of Ca^2+^ has nothing to do with the firmness of apples^3^. In addition, the firmness of cherry fruits regulated by exogenous Ca^2+^ is the same as that of apples regulated by exogenous Ca^2+^, which is also contradictory^15^. This is because the response of the fruit to Ca^2+^ is still unknown. Therefore, the application of Ca^2+^ imaging technology to study the relationship between the dynamic changes of Ca^2+^ in the cytoplasm and the physiological activities of pulp cells will help us to explore the regulatory role of Ca^2+^ in fruit firmness, soluble solids content, and physiological diseases in fruits, such as bitter pit.

In conclusion, we obtained viable protoplasts by enzymatic hydrolysis and then successfully loaded three small-molecule probes into the protoplasts at a high temperature (37 °C) for 30 min. The fluo-8/AM and fluo-4/AM fluorescence intensity was uniformly distributed in the cytoplasm of protoplasts and can be used at 5 μmol/L (Optimal Concentration) to determine the calcium concentration in the cytoplasm. In turn, rhod-2 was granulated in the cytoplasm and can be used to study calcium in the mitochondria. This method can provide technical support for calcium research in fruit and vegetable flesh tissue cells.

## Methods

### Production of flesh slices

We selected disease-free and mature ‘Fuji’ (*Malus domestica* Borkh. CV. Fuji) apples. The flesh tissue at 1–2 cm under the exocarp (skin) was cut with a scalpel. The flesh tissue was precooled in an embedding solution^57^, and 80 µm thick slices were cut with a microtome cryostat (Leica CM3050 S, Nussloch, Germany).

### Protocol for the extraction of single cells from apple flesh tissue

In accordance with our previously published method^58^, the flesh 1–2 cm below the exocarp was cut into small pieces of 1 × 1 × 1 mm^3^ and placed in a CPW (cell protoplast washing)^59^ solution containing 0.1% of macerozyme R-10 (w/v) at 28 °C and centrifuged at 70 rpm for 30 min in the dark. The enzyme solution was washed three times with CPW, stirred for 1 h with a magnetic stirrer, and centrifuged at 800 rpm for 3 min; the pulp with single cells was collected.

### Protoplast extraction protocols

The following basic solution was prepared to extract protoplasts: 20 mmol/L CaCl_2_·2H_2_O, 5 mmol/L MES, 0.6 mol/L D-sorbitol, and Tris buffer. The solution was adjusted to pH 5.8. The enzymatic solution was prepared from the following basic solution: 0.004 mg/ml macerozyme R-10 (Yakult, Japan), 0.0001 mg/ml pectolase Y-23 (Yakult, Japan), and 0.002% mg/ml cellulase R-10 (Yakult, Japan). The flesh tissue under the exocarp was cut into small pieces that were 10 × 5 × 1 mm^3^ in size (Fig. 2a), which were placed into 1.5 ml centrifuge tubes and added to 0.5 ml of the enzymatic solution. After the enzyme solution was digested at 28 °C for 1.5 h, it was immediately placed on ice to stop the reaction and then washed three times with a basic solution by centrifugation at 1000 rpm. Finally, the protoplast suspension was obtained.

### Protoplast viability assay

FDA (Fluorescein Diacetate, Thermo Fisher, USA) was dissolved in DMSO (Dimethyl Sulfoxide) to produce a 1 mg/ml stock solution. One microliter of stock solution was added to 99 μL of DMSO to prepare a working solution. Then, 99 μL of protoplast suspension was placed into a 0.2 ml centrifuge tube, and 1 μL of the FDA working solution was added. The staining was carried out for 5 min at 25 °C in the dark. Before observation, the stained sediments of protoplast suspensions were washed three times with basic solution by centrifugation at 1000 rpm. Then, the viability of the protoplasts was tested under a fluorescence microscope (EVOS Auto 2, Thermo Fisher, USA). We selected the light cube of GFP because the excitation wavelength of FDA is 490 nm^60^.

### Calcium ion fluorescence staining

Fluorescent loading solutions were prepared based on our previously published methods^43^. The concentration of the loading solution for preparing different kinds of fluorescent indicators was 0.5 mmol/L; a volume of 99 μL of sliced flesh tissue, single cell or protoplast suspension was placed into 0.5 ml centrifuge tubes, and 1 μL of a loading solution of fluo-4/AM, fluo-8/AM or rhod-2/AM (Dojindo Laboratories, Kumamoto, Japan) was added to make the final concentration of the fluorescent dye 5 µmol/L. We loaded the fluorescent dye into the cells for 30 min at 37 °C in the dark. After loading, the dye was washed three times with a basic solution to remove excess fluorescent dye and observed with a fluorescence microscope. Since the excitation wavelength of fluo-4/AM and fluo-8/AM is 490 nm, we selected GFP as the light cube. In turn, RFP was used as the light cube when loading rhod-2/AM because the excitation wavelength of rhod-2/AM is 551 nm.

### Measuring fluorescent trace

We took 18 μL of protoplasts after loading with fluo-8/AM, dropped it onto an adhesive on a concave microscope slide, covered the slide with a coverslip, and observed the calcium fluorescence change in the cells with a fluorescence microscope for 5 min. We then added 2 μL of EGTA (Ethylene Glycol-bis (beta-aminoethyl ether) -N,N,N′,N′-Tetraacetic Acid), A23187, ionomycin, or La^3+^ reagents so that their final concentrations were 10 mmol/L, 5 μmol/L, 1 μmol/L or 100 μmol/L, respectively. We then continued to observe the changes in calcium fluorescence for 25 min and took photographs every 5 min.

### Image analysis

The fluorescence results were analyzed using Image-Pro Plus 6.0 software (Media Cybernetics, Inc., MD, USA) according to our published methods^20^. For final processing, we used Adobe Photoshop CS5 (Adobe Systems, Mountain View, CA).

### Statistical analysis

Statistical analysis was performed using GraphPad Prism 7.0 software (GraphPad Software, Inc., La Jolla, CA, USA). Data are expressed as the means ± SD. Student’s *t*-test was used to analyze the differences among the experimental groups.

## Supplementary information

Supplementary figure S1

Supplementary figure S2

Supplementary figure S3

Supplementary figure S4

Supplementary figure S5

Supplementary figure S6

Supplementary figure S7

Supplementary figure S8

Supplementary information
