# Supplementary figures and images for: Loading calcium fluorescent probes into protoplasts to detect calcium in the flesh tissue cells of Malus domestica
Source: Hortic Res. 2020 Jun 1;7:91. doi: 10.1038/s41438-020-0315-3 (PMC7261807; doi:10.1038/s41438-020-0315-3)

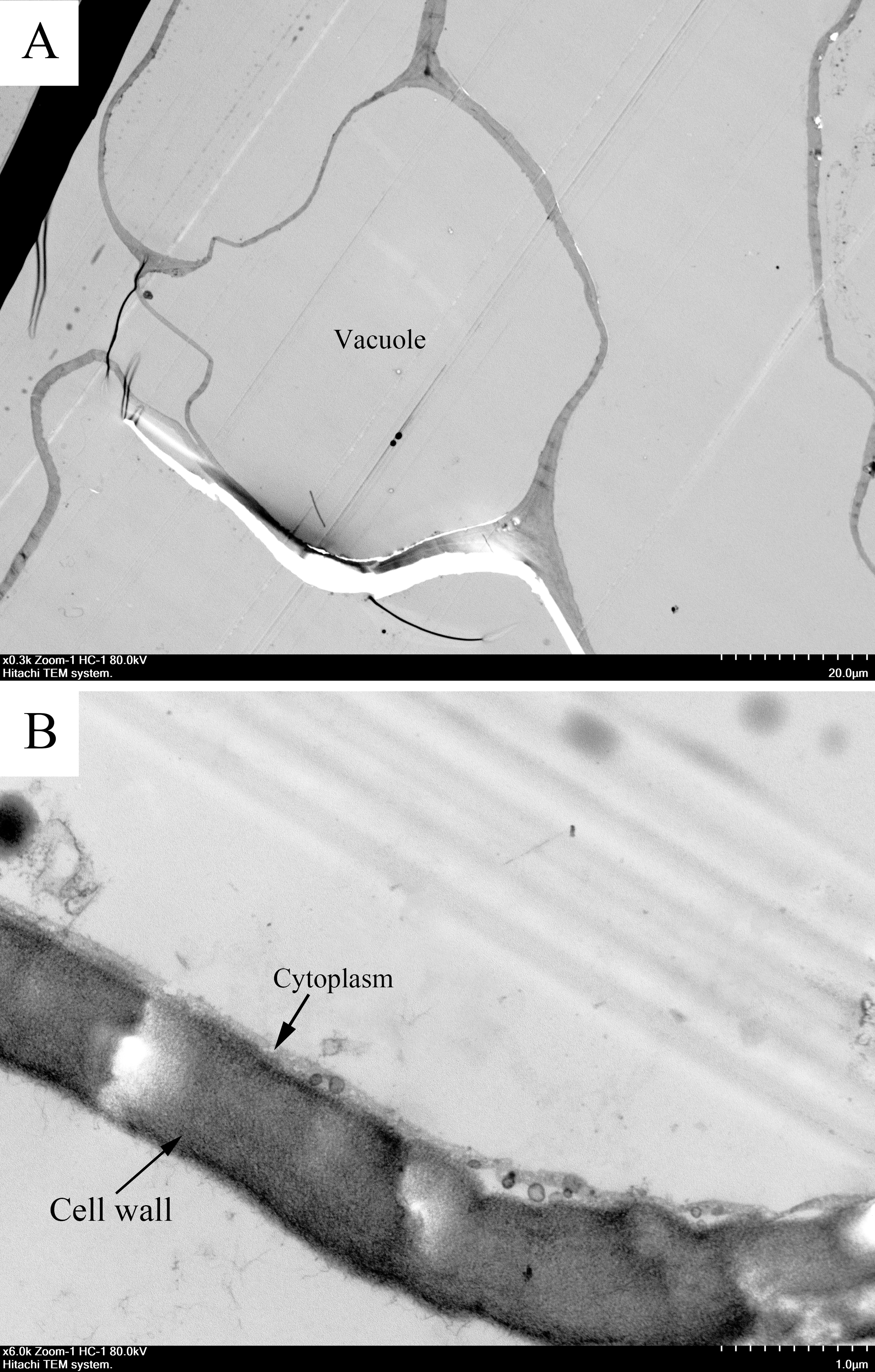

Supplement: Supplementary file 1 — Supplementary figure S1 [file 41438_2020_315_MOESM1_ESM.tif]

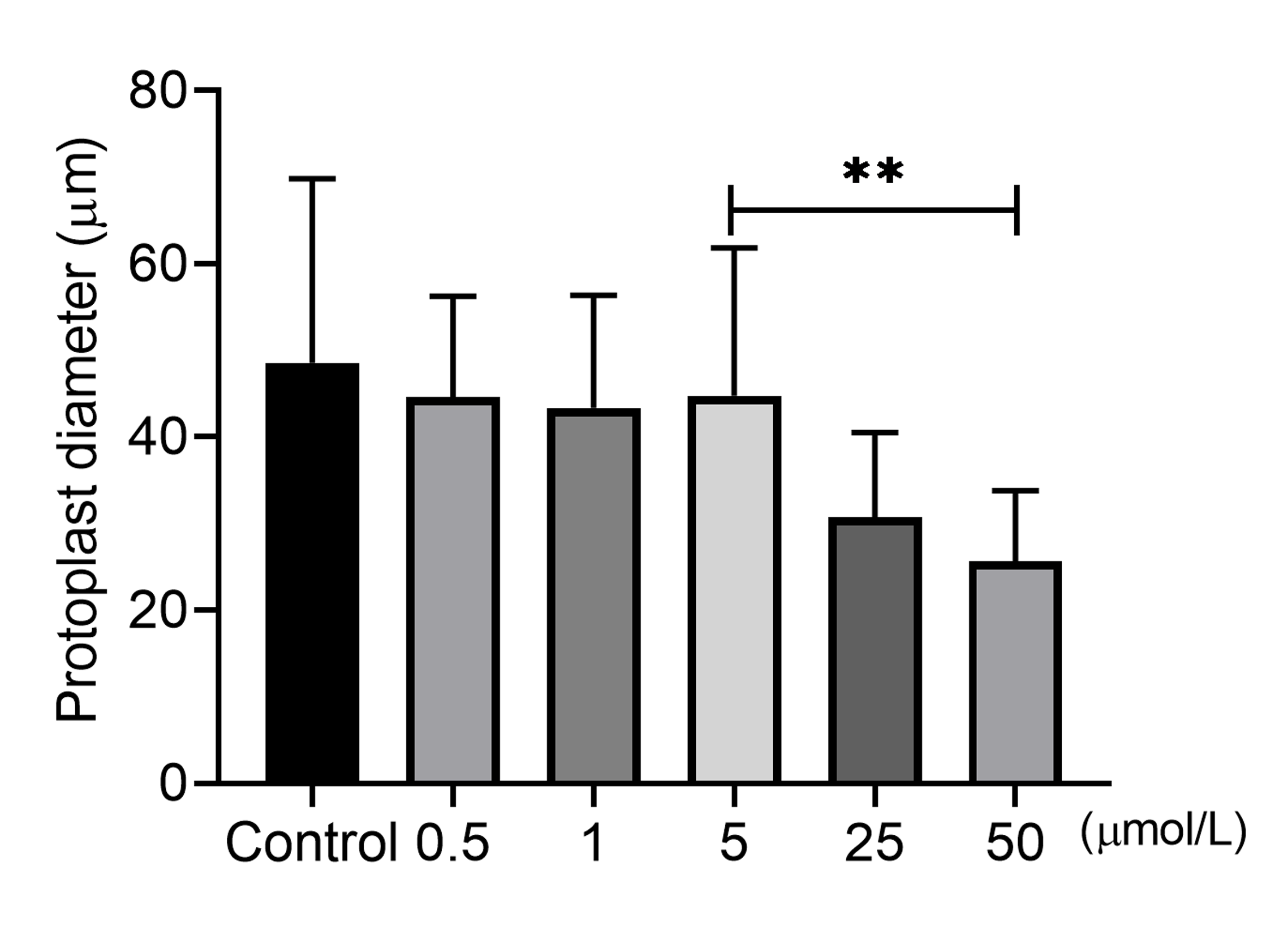

Supplement: Supplementary file 3 — Supplementary figure S3 [file 41438_2020_315_MOESM3_ESM.tif]

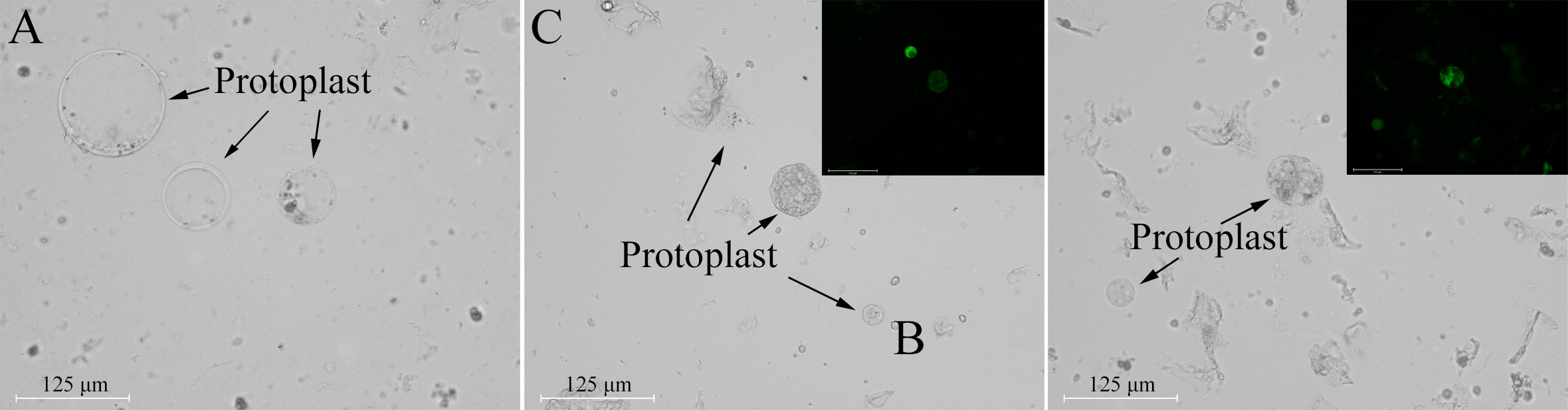

Supplement: Supplementary file 4 — Supplementary figure S4 [file 41438_2020_315_MOESM4_ESM.tif]

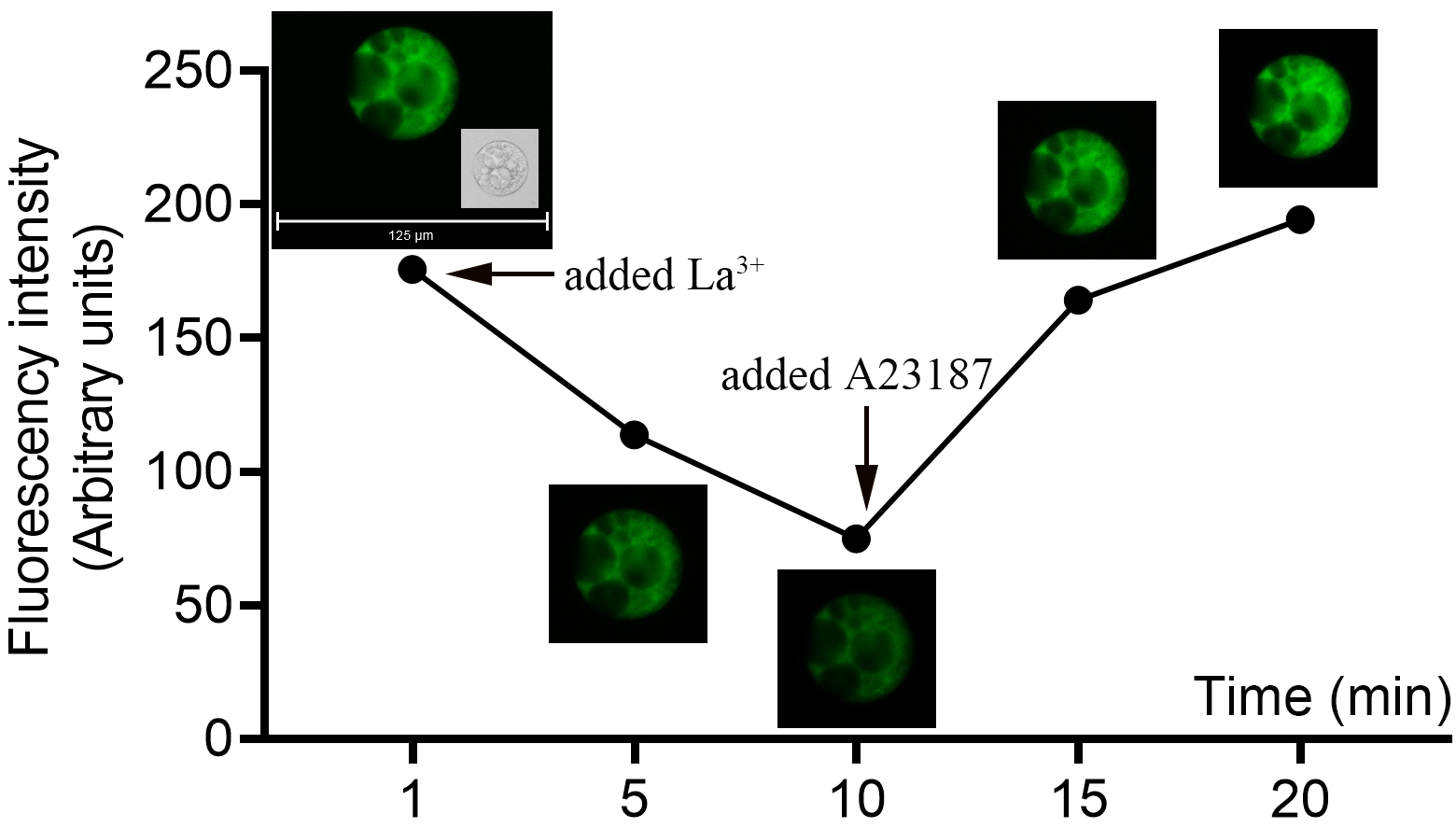

Supplement: Supplementary file 5 — Supplementary figure S5 [file 41438_2020_315_MOESM5_ESM.tif]

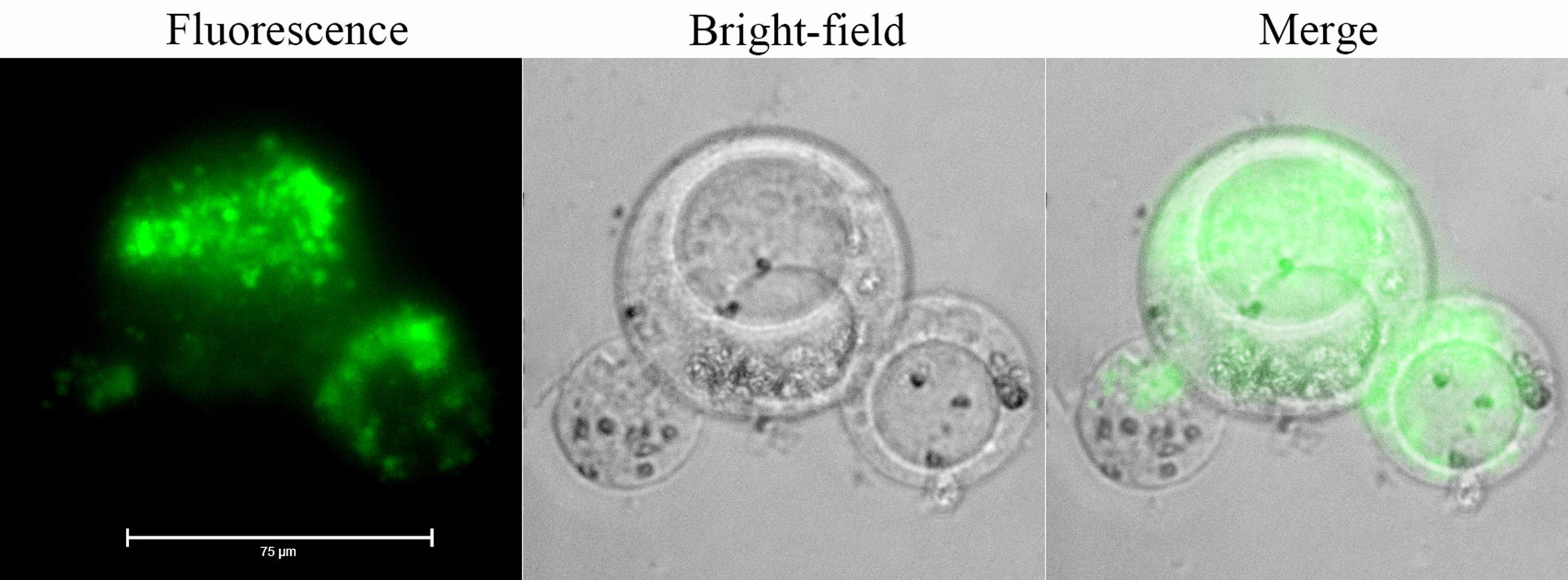

Supplement: Supplementary file 6 — Supplementary figure S6 [file 41438_2020_315_MOESM6_ESM.tif]

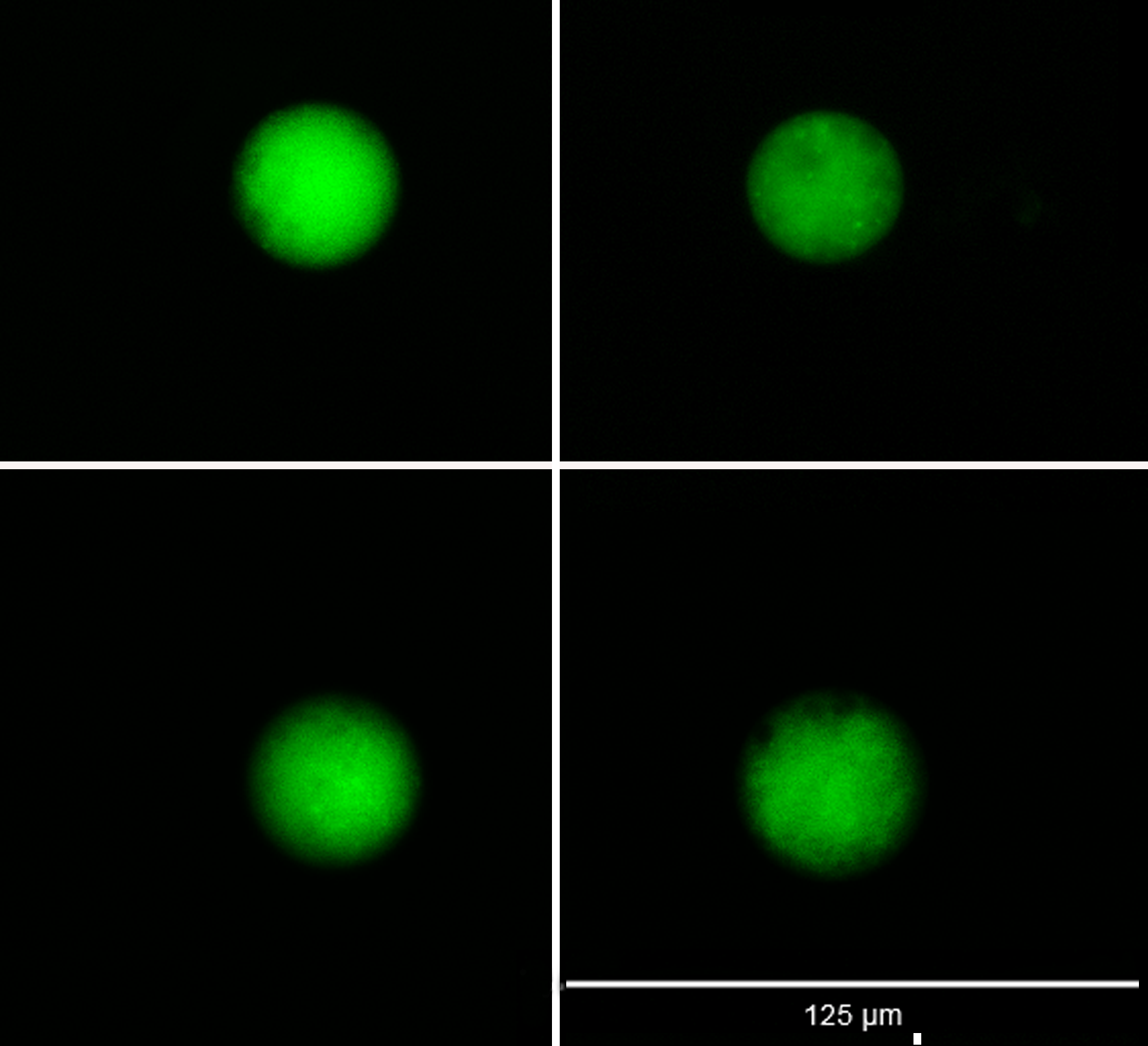

Supplement: Supplementary file 7 — Supplementary figure S7 [file 41438_2020_315_MOESM7_ESM.tif]

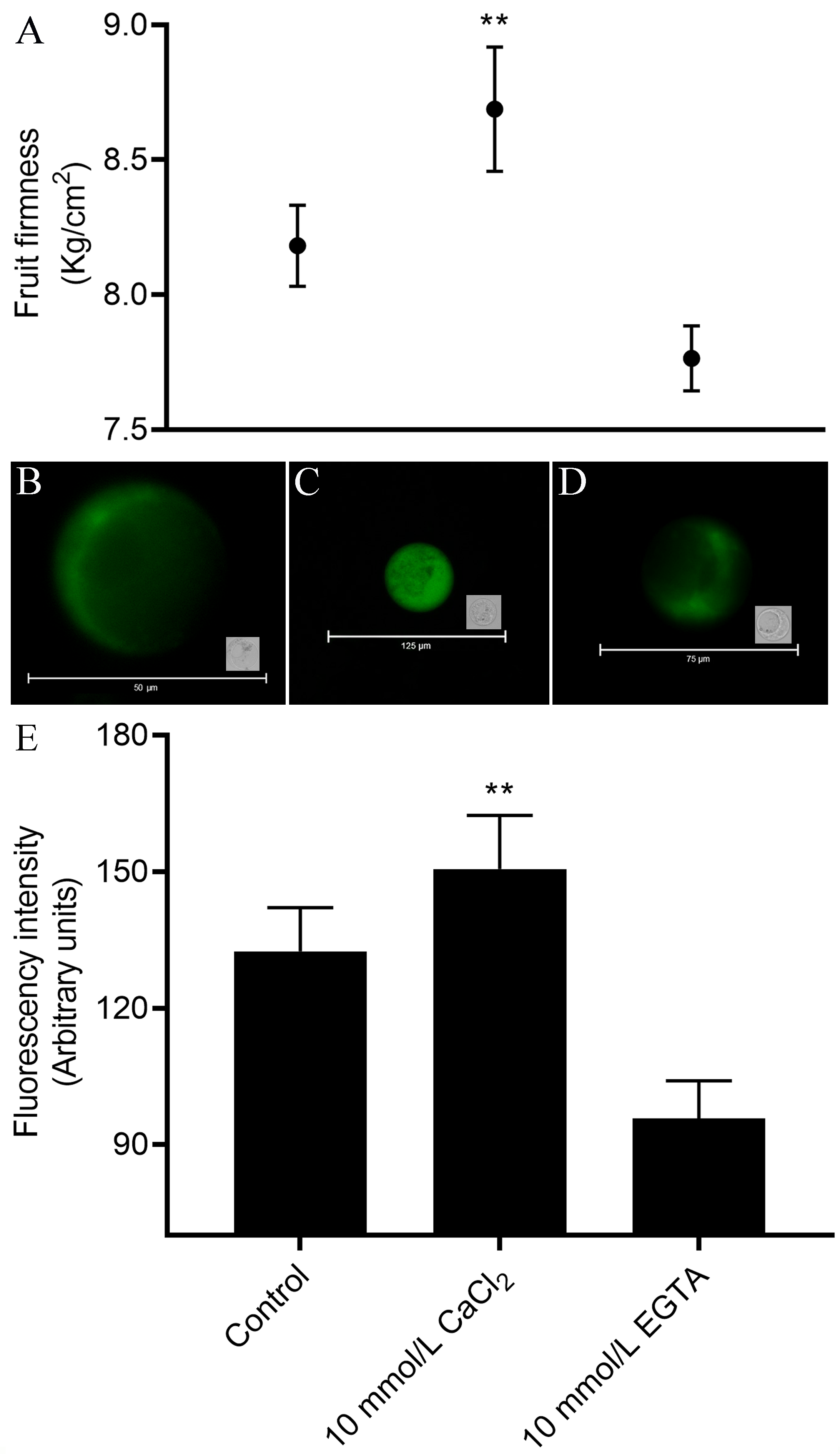

Supplement: Supplementary file 8 — Supplementary figure S8 [file 41438_2020_315_MOESM8_ESM.tif]
