# Supplementary material for: Loading calcium fluorescent probes into protoplasts to detect calcium in the flesh tissue cells of Malus domestica
Source: Hortic Res. 2020 Jun 1;7:91. doi: 10.1038/s41438-020-0315-3 (PMC7261807; doi:10.1038/s41438-020-0315-3)
Supplement: Supplementary file 9 — Supplementary information [file 41438_2020_315_MOESM9_ESM.docx]

**Supplementary Figure S1.** Two to three mm of fruit flesh tissue was drawn from under the exocarp (skin) and cut into 1 × 1 × 2 mm^3^ cuboids. The flesh tissue was fixed with 3% glutaraldehyde in 10 mmol/L PBS (phosphate-buffered saline), pH 7.2, for 2 h at room temperature, embedded into 2% agar and fixed in fresh fixatives under vacuum for 3 h. Flesh tissue for TEM observation was prepared according to a previously published method. ^57^ Ultrathin sections (60 nm) were cut with an ultramicrotome (Model: UC7, LEICA, Germany) and stained with uranyl acetate/lead citrate. Sections were examined using a Hitachi TEM system (Model: HT7700) at 80 kV. (A) A large vacuole in the center of the flesh tissue cells. (B) The vacuole pushes the cytoplasm around the cell against the cell wall. The arrow points to the cytoplasm.

**Supplementary Figure S2.** We measured the diameter of 50 protoplasts with Image-Pro Plus software. The diameters were counted using a histogram. The protoplast diameter ranged between 20 and 60 µm.

**Supplementary Figure S3.** Different concentrations of fluo-8/AM were loaded into the protoplasts for 30 min at 37°C. Then, we measured the diameter of protoplasts. When the concentration of fluo-8/AM exceeded 5 μmol/L, the diameter of the protoplasts was significantly reduced (*P* < 0.01). ** indicates a significant difference (*P* < 0.01, Student’s t-test).

**Supplementary Figure S4.** When the concentration of fluo-8/AM was 25 μmol/L and 50 μmol/L, the protoplasts shrunk, and their integrity was destroyed. (A) Control protoplasts were not loaded with fluo-8/AM. The protoplasts observed under the microscope were smooth and without deformation. (B) Protoplasts loaded with 25 μmol/L fluo-8/AM. (C) Protoplasts loaded with 50 μmol/L fluo-8/AM. In Fig. B and C, the protoplasts with larger diameters shrunk, while the protoplasts with smaller diameters did not shrink. The inset images in B and C are fluorescent images.

**Supplementary Figure S5.** We observed a change in the fluorescence intensity of Ca^2+^ in the same protoplast. After the protoplasts were loaded with fluorescence, Ca^2+^ fluorescence was observed, and then La^3+^ was added at a concentration of 100 µmol/L. The Ca^2+^ fluorescence intensity began to decrease. We added A23187 at a concentration of 5 µmol/L at 10 min. The fluorescence intensity of Ca^2+^ in the protoplast increased gradually.

**Supplementary Figure S6.** Fluo-8/AM was loaded into the protoplast at a low temperature (4°C) for 4 h. Although the fluorescent image shows fluorescence, this fluorescence was not found in the cytoplasm after the fluorescence image was merged with the bright field image. This result suggests that fluo-8/AM cannot be loaded into the protoplast at this low temperature (4°C).

**Supplementary Figure S7.** Fluo-8/AM loaded into the protoplast. We randomly selected four protoplasts without large vacuoles. Ca^2+^ fluorescence was uniformly distributed in these protoplasts.

**Supplementary Figure S8.** The relationship between the Ca^2+^ concentration in the cytoplasm of pulp cells and fruit firmness.

We chose ‘Golden Del. Reinders’ apple as the material because the fruit firmness of the variety decreases very quickly during maturity. Fifteen days before harvest, we gently scratched the fruit stalk with sandpaper (Model: 1500) on the apple tree and used filter paper to fully absorb 10 mmol/L Ca^2+^ or 10 mmol/L EGTA and wrapped the paper around the fruit stalk. We then wrapped the whole apple, including the stalk, in a black plastic bag. Filter paper was soaked with water and wrapped around the stalk as a control.

When the fruit was harvested, the firmness of the fruit was measured. The equatorial part of the fruit, the part near the stalk and the part near the sacral end were selected; then, four sites in each part were selected, the outermost layer (approximately 0.1 cm) of the peel was cut off, and the fruit firmness was measured with a fruit firmness tester (Model: GY-4), for which the pressure head diameter was 11 mm. The firmness value of each part of the fruit was the average value of the firmness of four sites, and a total of 30 fruits were tested.

(A) Firmness value of different treatments. A total of 30 apples were measured. Vertical bars indicate ± SE. ** indicates a significant difference (*P* < 0.01, Student’s t-test). (B) Control. (C) 10 mmol/L Ca^2+^. (D) 10 mmol/L EGTA. (E) Statistical analysis of fluorescence intensity in the protoplasts. ** indicates a significant difference (P < 0. 01, Student’s t-test). Vertical bars indicate ± SE. Each data point represents the mean of 6 protoplasts. (B-D) Representative fluorescence images of protoplasts.

**References**

57 Peng, W.-H., Lu, K.-S., Lai, S.-M., Shy, H.-T. & Kung, H.-N. Transmission Electron Microscopy (TEM) Protocol: Observation Details within Cells. *Bio-protocol* **3**, e816, doi:10.21769/BioProtoc.816 (2013).
